# Pan-Cancer Targeted Sequencing Reveals Genomic Heterogeneity and Prognostic Subgroups in Urothelial Bladder Cancer

**DOI:** 10.3390/cancers18061026

**Published:** 2026-03-22

**Authors:** Dimitar Ugrinovski, Skender Saidi, Viktor Stankov, Martina Ambardjieva, Slavica Josifovska, Anne-Katrin Koehler, Joerg Gabert, Sasho Panov

**Affiliations:** 1 Laboratory for Molecular Biology and Genomics, Institute of Biology, Faculty of Natural Sciences and Mathematics, Ss. Cyril and Methodius University, 1000 Skopje, North Macedonia; du@pathonext.de (D.U.); josifovskas@pmf.ukim.mk (S.J.); 2 PathoNext GmbH, 04103 Leipzig, Germany; ak@genolytic.de (A.-K.K.); jg@pathonext.de (J.G.); 3 University Clinic for Urology, Clinical Center, Medical Faculty, Ss. Cyril and Methodius University, 1000 Skopje, North Macedonia; skendersaidi@yahoo.com (S.S.); viktor_stankov@yahoo.com (V.S.); 4 PHI University Clinic for Surgical Diseases St. Naum Ohridski, Skopje, Medical Faculty, Ss. Cyril and Methodius University, 1000 Skopje, North Macedonia; martina.ambardzieva@gmail.com

**Keywords:** bladder cancer, urothelial carcinoma, next-generation sequencing, pan-cancer panel, *FGFR3*, *TP53*, *STAG2*, prognosis

## Abstract

**Background**: Urothelial bladder cancer (UBC) is a molecularly heterogeneous disease, and most sequencing studies have relied on bladder-specific or solid tumor-restricted panels. Whether broader pan-cancer assays provide additional clinically relevant information remains unclear. **Methods**: We performed targeted next-generation sequencing using an extended gene panel on tumor samples from 100 patients with UBC treated at a tertiary center. Somatic single-nucleotide variants, small insertions/deletions, copy-number alterations, and gene co-occurrence patterns were analyzed and correlated with clinicopathological features, disease-free survival (DFS), and overall survival (OS). **Results**: Recurrent alterations were identified in *FGFR3* (≈50%), *TP53* (≈35%), *STAG2* (≈25%), and *PIK3CA* (≈20%), consistent with established molecular pathways in UBC. Less frequent but potentially actionable alterations, including mutations in *BRCA1* and *ALK*, were also detected, reflecting the extended coverage of the panel. *TP53* mutations were independently associated with worse OS, whereas *STAG2* alterations were associated with improved OS, particularly in tumors without concurrent T*P53* mutations. *FGFR3* mutations showed a favorable but non-independent trend. No gene retained independent prognostic significance for DFS. Co-occurrence analysis revealed an *FGFR3/PIK3CA*-associated pathway and relative mutual exclusivity between *FGFR3* and *TP53*. Copy-number alterations were modest overall. Comparison with TCGA data demonstrated a higher frequency of *FGFR3* alterations in our cohort, likely reflecting the larger proportion of non–muscle-invasive tumors. **Conclusions**: Pan-cancer targeted sequencing provided a comprehensive genomic landscape of UBC, capturing canonical drivers and additional alterations that may be overlooked by bladder-restricted assays. The identification of *TP53* and *STAG2* as prognostic markers highlights the potential value of broader genomic profiling for biologically informed risk stratification in urothelial bladder cancer.

## Simple Summary

Bladder cancer shows substantial biological heterogeneity, and patients with similar clinical stages may experience markedly different outcomes. In this study, we analyzed tumor samples from 100 patients with urothelial bladder cancer using a broad targeted sequencing panel designed for pan-cancer testing. We confirmed that *TP53* mutations were associated with worse overall survival, whereas *STAG2* mutations were linked to a favorable prognosis. *FGFR3* mutations were frequent and more common in non–muscle-invasive tumors, although their prognostic impact was weaker after accounting for other clinical factors. Analysis of combined mutation patterns further highlighted distinct molecular subgroups. In addition to common bladder cancer drivers, we detected recurrent alterations in genes, such as *ERBB2* and *ATM*, which may have clinical relevance. In summary, our findings support the value of broader panel-based genomic profiling for identifying prognostic subgroups and better understanding molecular heterogeneity in real-world bladder cancer cohorts.

## 1. Introduction

Urothelial bladder carcinoma (UBC) is among the ten most frequently diagnosed malignancies worldwide and represents the fourth most common cancer among men in Western countries [1]. Based on invasion of the bladder muscle layer, UBC is broadly classified into non–muscle-invasive bladder cancer (NMIBC) and muscle-invasive bladder cancer (MIBC). Approximately half of patients with MIBC ultimately develop metastatic disease, with 5-year survival rates remaining around 50% despite intensive local and systemic therapies. Carcinoma in situ (CIS) represents a distinct subtype of NMIBC characterized by a flat, high-grade urothelial lesion with an increased risk of recurrence and progression [2]. Despite advances in surgical and intravesical therapies, disease recurrence is common, and a subset of patients with NMIBC will ultimately progress to muscle-invasive disease, contributing to poor long-term outcomes [3].

Recent genomic studies have demonstrated that UBC is characterized by marked molecular heterogeneity, with recurrent somatic alterations affecting pathways involved in chromatin regulation, cell-cycle control, receptor tyrosine kinase signaling, and DNA damage response. Both single-nucleotide variants and small insertions/deletions (SNVs/indels), as well as copy-number alterations (CNAs), have shown clinical relevance by informing risk stratification, refining molecular subtypes, and highlighting potentially actionable targets across disease stages.

These insights have supported the increasing use of targeted next-generation sequencing (NGS) panels in UBC research and clinical workflows, particularly in real-world settings where sequencing depth, cost, and turnaround time are critical considerations [4,5,6,7]. In addition, *TERT* promoter mutations represent one of the most frequent molecular events in UBC and have gained attention as highly specific urine-based biomarkers for early detection and disease monitoring, although their prognostic significance remains variable across cohorts [8,9].

Unlike most prior genomic studies that relied on bladder-specific or solid-tumor–restricted panels, we employed a broad pan-cancer targeted sequencing assay routinely used across diverse malignancies. This approach enabled us to assess whether extending gene coverage beyond conventional urothelial targets captures additional genomic alterations with potential biological, prognostic, or therapeutic relevance in urothelial bladder cancer.

## 2. Materials and Methods

### 2.1. Study Population and Sample Collection

Tumor specimens were obtained from 100 patients with urothelial bladder carcinoma (UBC) who underwent cystoscopy and transurethral resection of bladder tumor (TURBT) at the University Clinic for Urology in Skopje between January 2021 and August 2023 (hereafter referred to as the UBC100 cohort). Tumor samples were collected prospectively at the time of initial diagnosis, while clinical follow-up and outcome data were analyzed retrospectively. All enrolled patients were chemotherapy- and immunotherapy-naive at the time of sample collection. One patient presented with primary flat carcinoma in situ (pTis), a high-grade non–muscle-invasive lesion managed according to standard intravesical therapy protocols.

The primary outcomes of interest were overall survival (OS) and disease-free survival (DFS). Overall survival was defined as the time from initial diagnosis to death from any cause, with patients censored at the date of last follow-up. Disease-free survival was defined as the time from initial diagnosis to the first documented recurrence or metastasis, with patients censored at the date of last follow-up. Follow-up information was obtained from institutional medical records and outpatient documentation. Dates of diagnosis, recurrence, metastasis, last follow-up, and death were independently verified by at least two investigators.

Written informed consent was obtained from all patients, and the study was approved by the Ethics Committee of the University Clinic of Urology (approval number 03-16/2, 4 January 2021). Tissue samples were collected during the initial TURBT procedure, snap-frozen, and stored at −80 °C. Only specimens with histopathologically confirmed UBC were included.

### 2.2. DNA Extraction

Genomic DNA was extracted from RNAlater-preserved tumor tissue using the DNA Tissue Extraction Kit (#RTD291) and the Nextractor 48S System, both from Genolution, Seoul, Republic of Korea, according to the manufacturer’s instructions. DNA concentration was measured using a Qubit 3.0 fluorometer with the Qubit DNA Broad Range Assay Kit, both from Thermo Fisher Scientific, Waltham, MA, USA. DNA quality was assessed using the E-Gel Power Snap electrophoresis system (Thermo Fisher Scientific, Waltham, MA, USA). Only samples with an mean fragment length > 500 bp and DNA concentration > 50 ng/µL were included in subsequent analyses.

### 2.3. Targeted Sequencing Panel

Samples were analyzed using a custom-designed pan-cancer targeted sequencing panel covering 95 genes (Supplementary Table S1). The panel was designed for mutational analysis of solid and hematological malignancies and targeted recurrent mutational hotspots, selected exons and introns, as well as the *TERT* promoter region, spanning a total target size of approximately 204 kb.

### 2.4. Library Preparation and Next-Generation Sequencing (NGS)

A total of 50 ng of genomic DNA was used for library preparation using the KAPA EvoPlus v2 Kit (Roche Sequencing Solutions, Basel, Switzerland). Following adapter ligation, pre-capture PCR amplification was performed using KAPA HiFi HotStart ReadyMix (Roche Sequencing Solutions, Basel, Switzerland) according to the manufacturer’s instructions. Libraries were evaluated on a TapeStation 4200 using the D1000 ScreenTape Assay (Agilent Technologies, Santa Clara, CA, USA). Target enrichment was performed using hybrid capture with a custom probe panel, after which libraries were purified and assessed for fragment size distribution and DNA concentration using TapeStation 4200 and Qubit 3.0 fluorometer. Sequencing was performed on the Illumina NovaSeq 6000 platform (Illumina, San Diego, CA, USA) using a NovaSeq SP flow cell in paired-end 2 × 150 bp mode. Real-time quality control was monitored using Illumina Sequencing Analysis Viewer (Illumina, San Diego, CA, USA). The mean sequencing depth exceeded 1000×, and more than 92% of bases achieved a Phred quality score ≥ Q30. Demultiplexing was performed using bcl2fastq (v2.20, Illumina, San Diego, CA, USA), and reads were aligned to the human reference genome (GRCh37/hg19) using CLC Genomics Workbench (Qiagen, Aarhus, Denmark).

### 2.5. Variant Calling and Classification

Variant calling was performed using CLC Genomics Workbench (v24). The following filtering criteria were applied: minimum read depth of 200×, variant allele frequency (VAF) ≥ 3% for SNVs and ≥5% for indels, and strand-bias filtering excluding variants with >80% of supporting reads derived from a single strand. Variants were annotated and prioritized using CLC Genomics Workbench and assessed against COSMIC, ClinVar, dbSNP, and gnomAD databases. Additional annotation and clinical interpretation support were obtained using Franklin/Genoox (https://franklin.genoox.com), VarSome (https://varsome.com), and GeneBe (https://genebe.net), all accessed in January 2026. Manual review of read alignments and local sequence context was performed using Integrative Genomics Viewer (IGV v2.18.4) [10].

Variants were classified according to clinical significance as pathogenic, likely pathogenic, or variants of uncertain significance (P/LP/VUS) and were used for downstream analyses. ClinVar presence was evaluated separately using coordinate-based matching against the ClinVar GRCh37 (hg19) VCF. Because sequencing was performed on tumor tissue without matched normal samples, detected variants represent alterations observed in tumor-only sequencing, and a germline origin cannot be definitively excluded for individual variants. Population allele frequency data and clinical annotation databases were used to support variant interpretation, but confirmatory germline testing would be required to establish hereditary origin when clinically indicated. Copy-number alterations were inferred from normalized read-depth data generated by the targeted sequencing panel and were interpreted at the gene level.

### 2.6. Statistical Analysis

Statistical analyses were performed in R version 4.4.2 (R Foundation for Statistical Computing, Vienna, Austria). The packages maftools (version 2.22.0) and MutationalPatterns (version 3.16.0) were used for mutational analysis and visualization [11,12]. Associations between clinicopathological variables and mutation status were evaluated using two-sided Fisher’s exact tests, with odds ratios (OR) and 95% confidence intervals (CI) reported. To account for multiple testing in gene–phenotype association analyses, *p*-values were adjusted using the Benjamini–Hochberg false discovery rate (FDR) method, and the resulting q-values are reported alongside the original *p*-values. Multivariable logistic regression models were fitted for selected clinicopathological endpoints, including mutation status of recurrently altered genes as predictors and adjusting for age and sex. Model performance was assessed using receiver operating characteristic (ROC) curves and summarized by the area under the curve (AUC). Survival analyses were performed using Kaplan–Meier estimates and Cox proportional hazards models. Two-sided *p*-values < 0.05 were considered statistically significant.

## 3. Results

### 3.1. Clinicopathological Characteristics of the Patients

The demographic and clinicopathological characteristics of the 100 patients included in this study are summarized in Table 1. The UBC100 cohort was predominantly male (78%) with a mean age of 66.6 years (range, 41–86). At initial diagnosis, two-thirds of patients presented with non–muscle-invasive bladder cancer (NMIBC), whereas one-third had muscle-invasive disease (MIBC). High-grade tumors were observed in 70% of cases, and unifocal tumors larger than 3 cm were present in the majority of patients. A single case presented as primary carcinoma in situ (CIS).

Approximately 56% of patients reported a moderate or heavy smoking history. The mean observed disease-free survival (DFS) and overall survival (OS) times were 22.21 and 25.68 months, respectively, reflecting follow-up duration under administrative censoring. The median follow-up time, defined as the interval from initial TURBT to death or last follow-up, was 24.5 months (range, 2.0–53.0 months), with administrative censoring in July 2025. Imaging-based evidence of distant metastasis confirmed by CT and/or MRI was documented in seven patients. In addition, radical cystectomy was performed in 27 patients with muscle-invasive bladder cancer as part of standard clinical management. Taken together, substantial clinical heterogeneity was observed across the cohort, providing the clinical context for subsequent genomic and outcome analyses.

### 3.2. Genetic Alterations Identified by Targeted Sequencing

Targeted sequencing of 100 urothelial bladder cancer tumors using a 95-gene pan-cancer panel identified somatic variants in all samples. Variant annotation across the cohort identified 248 pathogenic, 36 likely pathogenic, 161 variants of uncertain significance, 65 likely benign, and 84 benign variants. After exclusion of likely benign and benign variants, a total of 445 pathogenic, likely pathogenic, or variants of uncertain significance (P/LP/VUS) were retained for downstream analyses. In summary, 66 of the 95 genes included in the panel harbored at least one P/LP/VUS alteration across the cohort.

**Figure 1:**
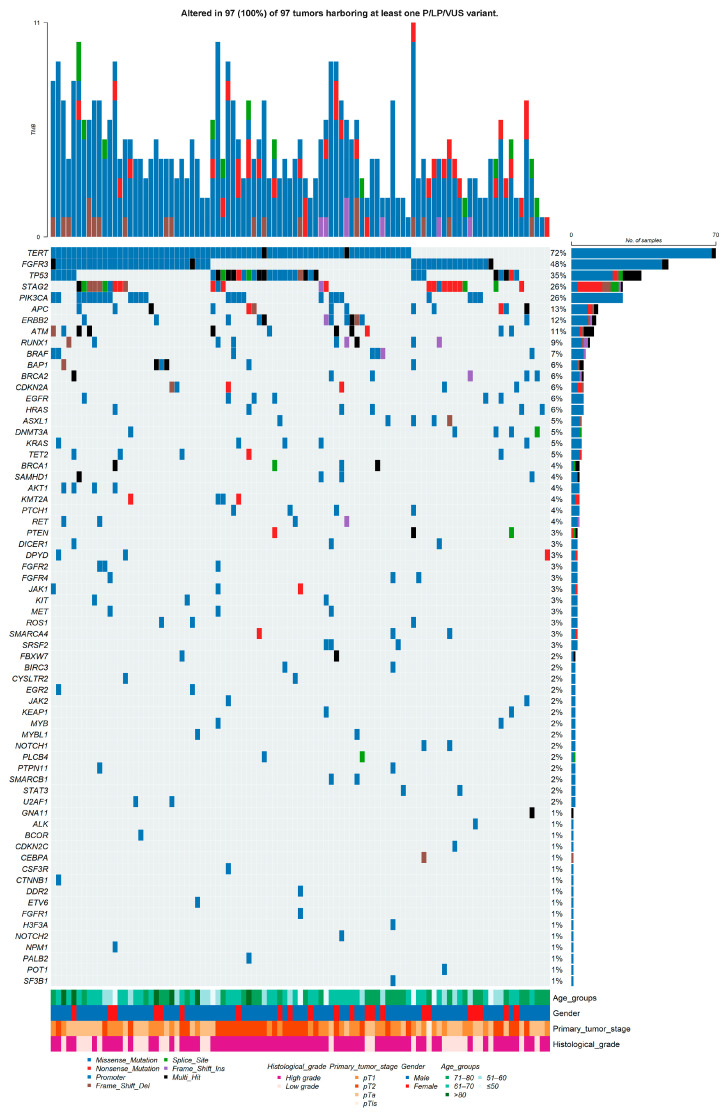
Somatic mutation landscape of the UBC100 cohort. An oncoplot summarizing pathogenic/likely pathogenic (P/LP) and variants of uncertain significance (VUS) across 100 urothelial bladder cancer tumors profiled using a 95-gene targeted sequencing panel. The plot displays non-synonymous SNVs/indels and *TERT* promoter mutations (C228T and C250T), whereas copy-number alterations are excluded and presented separately. Genes are ordered by overall mutation frequency, and tumors are arranged to highlight recurrent patterns and clustering among frequently altered genes. Each column represents one tumor sample, and each row represents one gene. Multi_Hit indicates genes harboring more than one alteration within the same tumor. Color codes for variant classes and clinical annotation tracks (age group, sex, smoking history, pathological stage, and histological grade) are shown below the oncoplot. Percentages displayed in the oncoplot are rounded for visualization; exact values are provided in the text.

The overall somatic mutation landscape of the cohort is summarized in Figure 1, which displays non-synonymous SNVs/indels and *TERT* promoter mutations across tumors harboring at least one P/LP/VUS alteration (*n* = 97).

The targeted sequencing panel used in this study included genes representing multiple oncogenic pathways beyond canonical urothelial driver genes. A comparison of gene and pathway coverage between representative bladder-focused sequencing panels reported in the literature and the broader pan-cancer panel used in this study is provided in Supplementary Table S2.

*TERT* promoter alterations represented the most frequent genomic event, detected in 70 of 97 tumors (72.16%), followed by recurrent mutations in established bladder cancer genes, including *FGFR3* (49.48%), *TP53* (36.08%), *STAG2* (26.80%), *PIK3CA* (25.77%), and *ATM* (21.65%). Additional recurrently altered genes included *BRCA2* (19.59%), *APC* (17.53%), and *ERBB2* (15.46%), while *KMT2A* remained among the ten most frequently mutated genes (11.34%).

The oncoplot reveals distinct mutational patterns, with subsets of tumors dominated by *FGFR3*-associated alterations and others enriched for *TP53*-related mutations, consistent with divergent molecular routes of urothelial carcinogenesis. Clinical annotation tracks demonstrate that these genomic patterns spanned a broad range of clinicopathological characteristics, underscoring the biological heterogeneity of the cohort.

Among the most frequently altered genes, *FGFR3* mutations were dominated by recurrent activating hotspot missense variants, particularly p.Ser249Cys and p.Tyr373Cys, which were observed together in 27 tumors. In contrast, *TP53* alterations comprised a mixture of missense substitutions and truncating variants, with p.Arg175His and p.Arg213* being the most frequent. *STAG2* mutations were characterized predominantly by loss-of-function events, including nonsense and frameshift variants, consistent with tumor suppressor inactivation. *PIK3CA* mutations clustered at canonical hotspot positions (p.Glu545Lys, p.Glu542Lys, and p.His1047Arg), collectively affecting 22 tumors, with no tumor harboring more than one hotspot mutation. Detailed variant-level information is provided in Supplementary Table S3.

Across tumors harboring at least one P/LP/VUS alteration, the number of detected somatic variants per tumor ranged from 1 to 11, with a median of 5 variants (interquartile range, 3–6), reflecting substantial intertumoral variability within the targeted genomic space.

Notably, the single case of primary flat carcinoma in situ (pTis) harbored multiple pathogenic driver alterations, including mutations in *FGFR3*, *PIK3CA*, and *STAG2*, illustrating that early non-invasive lesions may already carry complex oncogenic profiles detectable by next-generation sequencing.

Coordinate-based matching against ClinVar (GRCh37/hg19) revealed that a subset of P/LP/VUS alterations lacked existing ClinVar annotation. Manual curation of selected variants in recurrently altered genes confirmed that several remained unreported in ClinVar at the time of analysis (Table 2), highlighting the importance of variant-level verification when inferring rarity or novelty from automated annotation pipelines.

Finally, a subset of somatic alterations identified in the cohort fulfilled established criteria for potential clinical actionability. These variants, including selected single-nucleotide variants and copy-number alterations, were classified according to AMP/ASCO/CAP joint consensus guidelines. Alterations in *FGFR3*, *PIK3CA*, and *ERBB2* reached at least Tier II levels of evidence in a substantial proportion of tumors and, when considered cumulatively, were present in up to 81% of patients in the cohort.

Corresponding approved or investigational targeted therapies are summarized in Supplementary Table S4.

### 3.3. Mutation Spectrum and Substitution Patterns

**Figure 2:**
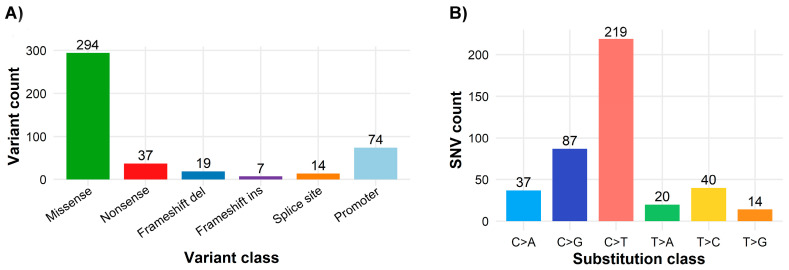
Mutational spectrum of pathogenic, likely pathogenic, and variants of uncertain significance (P/LP/VUS) in the UBC100 cohort. (**A**) Distribution of somatic variants by functional class, including missense, nonsense, frameshift insertions and deletions, splice-site, and promoter variants. Only variants annotated as pathogenic, likely pathogenic, or variants of uncertain significance were included. (**B**) Somatic single-nucleotide variant (SNV) substitution spectrum showing pyrimidine-normalized base substitution classes (C>A, C>G, C>T, T>A, T>C, and T>G) derived from the same filtered variant set.

Across the 97 tumors harboring at least one pathogenic, likely pathogenic, or variant of uncertain significance (P/LP/VUS) alteration, a total of 489 somatic single-nucleotide variants (SNVs) were identified within the targeted gene panel. The distribution of functional variant classes was dominated by missense substitutions, with smaller contributions from nonsense, frameshift, splice-site, and promoter variants (Figure 2A).

Analysis of the SNV substitution spectrum revealed a strong predominance of C>T transitions, followed by C>G and T>C substitutions, whereas C>A, T>A, and T>G changes were relatively infrequent (Figure 2B). Overall, transition events were more common than transversions, consistent with previously reported mutational patterns in urothelial carcinoma. The median per-sample transition/transversion (Ti/Tv) ratio was 1.0 (interquartile range, 0.6–2.0; range, 0–13.0), reflecting intertumoral heterogeneity and the constrained genomic scope of the targeted sequencing panel.

### 3.4. Copy-Number Alteration Landscape

**Figure 3:**
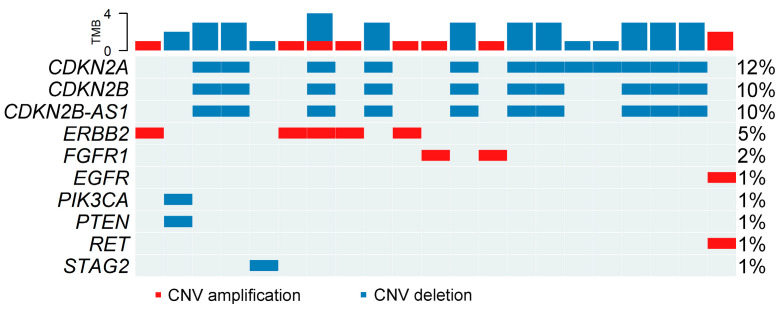
Copy-number alterations in selected genes. CNV oncoplot showing amplifications (red) and deletions (blue) across key cancer-related genes in the UBC100 cohort. Samples are ordered according to single-nucleotide variant burden for consistency with Figure 1. For visualization clarity, only samples with at least one copy-number alteration (amplification or deletion) are shown, whereas reported frequencies are calculated across the entire cohort (*n* = 100).

Copy-number changes were detected in 45 of 100 tumors (45%), largely restricted to a limited number of recurrently affected loci. The most frequent events involved deletions in the 9p21 region affecting *CDKN2A/CDKN2B* and focal amplifications in genes associated with receptor tyrosine kinase signaling, including *ERBB2*, *EGFR*, and *FGFR1* (Figure 3). Although the CNV oncoplot visualizes only tumors harboring at least one copy-number alteration, all reported frequencies refer to the entire cohort.

These alterations were largely mutually exclusive with point mutations affecting the same pathways, and were more frequently observed in muscle-invasive tumors. Notably, *STAG2* deletions were uncommon, consistent with its predominant inactivation through sequence-level truncating mutations rather than copy-number loss. Collectively, these findings underscore the biological heterogeneity of urothelial bladder cancer and highlight the coexistence of pathway-specific sequence-level and copy-number alterations within the cohort. No copy-number alterations were observed in the single carcinoma in situ (CIS) case, despite the presence of multiple pathogenic point mutations.

### 3.5. The Association Between Genotype and Clinical Characteristics

**Figure 4:**
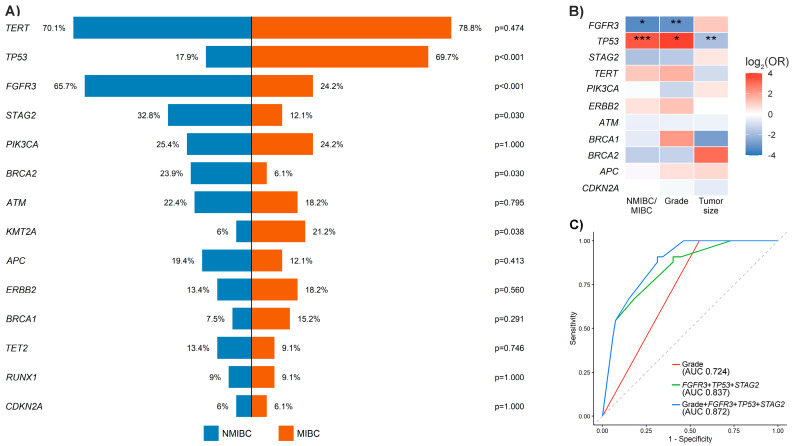
Genotype–phenotype associations and discriminative value of genomic alterations in the UBC100 cohort. (**A**) Gene-level comparison of somatic mutation frequencies between non–muscle-invasive bladder cancer (NMIBC, blue) and muscle-invasive bladder cancer (MIBC, orange). Bars represent the percentage of tumors harboring at least one qualifying somatic variant per gene; *p*-values were calculated using Fisher’s exact test. (**B**) Heatmap summarizing associations between selected gene mutations and clinicopathological features, including invasiveness (NMIBC/MIBC), histological grade, tumor stage, and tumor size. Colors indicate the direction and magnitude of association expressed as log_2_ odds ratios (red, enrichment; blue, depletion). Statistical significance indicators correspond to associations that remained significant after Benjamini–Hochberg correction for multiple testing (* *q* < 0.05, ** *q* < 0.01, *** *q* < 0.001). (**C**) Receiver operating characteristic (ROC) curves evaluating the ability of histological grade alone, recurrent genomic alterations (*FGFR3*, *TP53*, and *STAG2*), and their combination to discriminate MIBC from NMIBC (AUC, area under the curve). The diagonal dashed line indicates random classification performance (AUC = 0.5).

Comparison of gene-level somatic mutation frequencies between NMIBC and MIBC revealed distinct patterns of genomic alteration within the UBC100 cohort (Figure 4A). Mutations in *FGFR3* and *STAG2* were more prevalent in NMIBC, whereas TP53 alterations and mutations affecting several DNA damage–repair (DDR) genes, including *ATM* and *BRCA1*, were more frequent in MIBC. Fisher’s exact test demonstrated significant depletion of *TP53* mutations in NMIBC compared with MIBC (17.9% vs. 69.7%; OR = 0.108, 95% CI 0.036–0.300; *p* = 1.54 × 10^−6^), as well as in low-grade compared with high-grade carcinomas (17% vs. 44%; OR = 0.127, 95% CI 0.023–0.470; *p* = 2.75 × 10^−4^). In contrast, *FGFR3* mutations predominated in NMIBC compared with MIBC (65.7% vs. 24.2%; OR = 5.01, 95% CI 1.88–14.43; *p* = 5.43 × 10^−4^) and were significantly more frequent in low-grade than in high-grade tumors (83% vs. 35%; OR = 7.35, 95% CI 2.39–27.56; *p* = 6.79 × 10^−5^).

The strongest gene–clinical associations involved *FGFR3* and *TP53*, which exhibited strikingly opposite distributions across major clinicopathological parameters (Figure 4B). To account for potential confounding, selected genotype–phenotype associations were further evaluated using age- and sex-adjusted logistic regression models (Figure 4C). Receiver operating characteristic (ROC) analysis demonstrated that recurrent genomic alterations (*FGFR3*, *TP53*, and *STAG2*) discriminated MIBC from NMIBC more accurately than histological grade alone, while a combined clinical–genomic model achieved the highest discriminatory performance. These findings indicate that genomic profiling provides prognostic information complementary to conventional pathological assessment. Detailed association results for recurrent genomic alterations (*TERT*, *FGFR3*, *TP53*, and *STAG2*) are presented in Table 3. Recurrence events were observed exclusively in NMIBC cases, consistent with standard clinical management.

**Figure 5:**
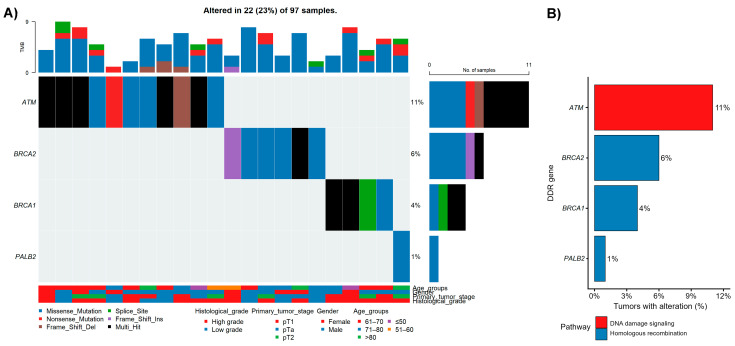
Landscape and frequency of DNA damage–repair (DDR) gene alterations in the UBC100 cohort. (**A**) Oncoplot summarizing pathogenic (P), likely pathogenic (LP), and variants of uncertain significance (VUS) somatic alterations in selected DDR genes across 97 clinically annotated bladder cancer samples harboring at least one qualifying variant, together with key clinicopathological annotations. Mutation types are color-coded (missense, nonsense, frameshift, splice-site, and multi-hit) as in Figure 1. (**B**) Gene-level frequencies of DDR alterations, grouped by major functional pathways. Percentages indicate the proportion of tumors harboring at least one qualifying alteration per gene (denominator = 97).

Given the central role of DNA damage–repair (DDR) pathways in bladder cancer biology, genomic instability, and therapeutic response, we next examined alterations affecting core DDR genes within the UBC100 cohort. Among the 97 tumors harboring at least one pathogenic, likely pathogenic, or VUS alteration, DDR gene alterations were detected in 22 tumors (23%) (Figure 5). Alterations most frequently involved *ATM* (11.34%), followed by *BRCA2* (6.19%), *BRCA1* (4.12%), and *PALB2* (1.03%). These alterations were not uniformly distributed but clustered within specific functional DDR pathways, particularly DNA damage signaling and homologous recombination, rather than reflecting a generalized increase in mutational burden. Collectively, these findings highlight a subset of tumors characterized by recurrent, pathway-specific DDR gene alterations.

Because tumor-only targeted sequencing may occasionally detect variants arising from age-related clonal hematopoiesis, an exploratory analysis focusing on CHIP-associated genes was performed. Logistic regression showed no significant association between age and the presence of CHIP-associated alterations (OR per 10 years = 1.03; 95% CI 0.66–1.59; *p* = 0.888). In addition, low-variant allele fraction events were uncommon among tumors harboring CHIP-associated variants (VAF < 5% in 2 of 19 tumors, ~10.5%), consistent with most alterations occurring at moderate-to-high allele fractions.

### 3.6. Gene-to-Gene Interaction Analysis

**Figure 6:**
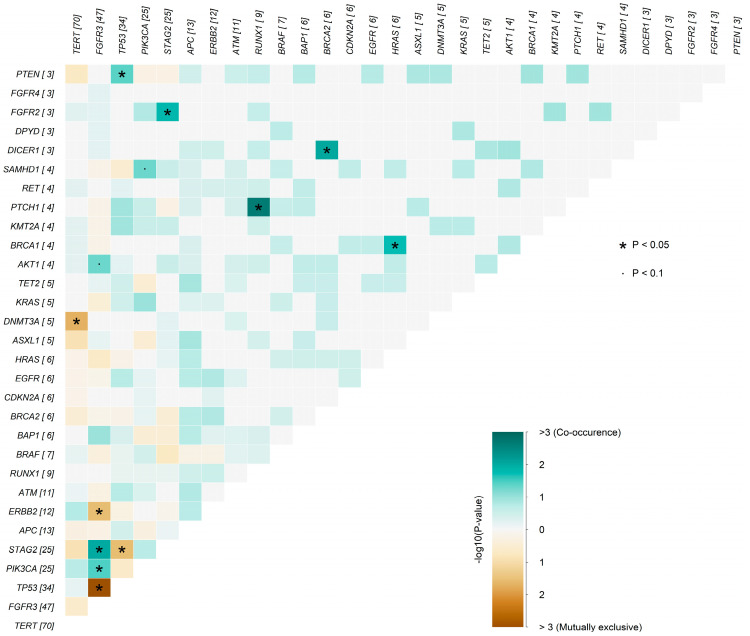
Somatic interaction analysis of frequently altered genes in the UBC100 cohort. Co-occurrence and mutual exclusivity patterns were assessed for the 30 most frequently mutated genes. Significant gene–gene relationships are displayed as a pairwise interaction matrix, in which positive associations indicate co-occurrence and negative associations indicate mutual exclusivity. The strongest signal of co-occurrence was observed between *FGFR3* and *PIK3CA*, whereas *FGFR3* and *TP53* showed a prominent pattern of mutual exclusivity. A weaker trend toward co-occurrence between *FGFR3* and *STAG2* was also observed. The color scale represents −log_10_ (*p*-value), with green tones indicating co-occurrence and brown tones indicating mutual exclusivity; statistically significant pairs (*p* < 0.05) are annotated with (*). Numbers in brackets indicate the number of samples harboring alterations in each gene.

Gene-to-gene interaction analysis identified distinct patterns of co-occurrence and mutual exclusivity among recurrently altered genes in the UBC100 cohort (Figure 6). *FGFR3* mutations frequently co-occurred with *PIK3CA* alterations, whereas *TP53* mutations exhibited a pronounced pattern of mutual exclusivity with *FGFR3*, consistent with biologically divergent molecular programs. A similar trend toward co-occurrence between *FGFR3* and *STAG2* was also observed, although this association did not reach formal statistical significance. Collectively, these interaction patterns suggest the presence of at least two major molecular trajectories within the cohort: an *FGFR3*/PI3K-driven pathway, typically associated with non–muscle-invasive disease, and a *TP53*-driven pathway enriched in more aggressive tumors.

### 3.7. Survival Results

In survival analyses, *TP53* mutations were associated with significantly worse overall survival (OS), whereas *STAG2* alterations showed a protective effect. *FGFR3* mutations were associated with a trend toward improved OS but did not reach statistical significance in multivariable models.

In univariable Cox proportional hazards analyses, *TP53* mutations were associated with significantly worse OS (HR = 2.61, 95% CI 1.36–5.00; *p* = 0.0038), whereas *STAG2* mutations were strongly associated with improved OS (HR = 0.18, 95% CI 0.05–0.58; *p* = 0.0041). *FGFR3*-mutated tumors showed a non-significant trend toward improved survival (HR = 0.55, 95% CI 0.28–1.05; *p* = 0.069). After adjustment for age and gender, *TP53* remained an adverse prognostic factor (HR = 2.79, 95% CI 1.45–5.37; *p* = 0.0021), and *STAG2* remained significantly protective (HR = 0.20, 95% CI 0.06–0.67; *p* = 0.0091), whereas the effect of *FGFR3* did not reach statistical significance (HR = 0.57, 95% CI 0.29–1.10; *p* = 0.095). In a combined multivariable model including *TP53*, *FGFR3*, *STAG2*, age, and gender, both *TP53* (HR = 2.16, 95% CI 1.11–4.22; *p* = 0.023) and *STAG2* (HR = 0.29, 95% CI 0.08–0.98; *p* = 0.046) retained independent prognostic value, whereas *FGFR3* mutation status did not (HR = 0.71, 95% CI 0.37–1.39; *p* = 0.323).

**Figure 7:**
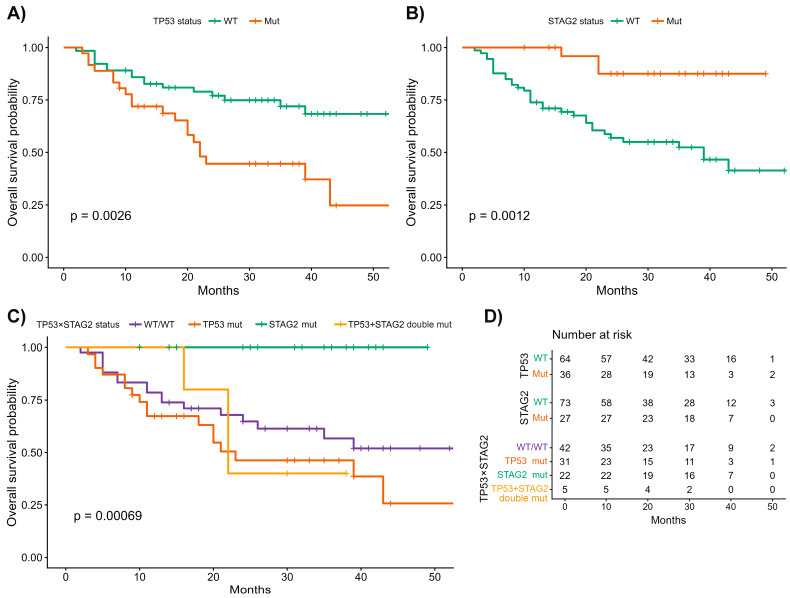
Kaplan–Meier overall survival according to *TP53* and *STAG2* mutational status. (**A**) Patients with *TP53*-mutated tumors exhibited significantly worse overall survival compared with those with *TP53* wild-type tumors. (**B**) *STAG2*-mutated tumors were associated with markedly improved overall survival. (**C**) Combined *TP53*/*STAG2* stratification demonstrated the poorest outcomes in the *TP53*-mutant/*STAG2*-wild-type group, whereas no deaths occurred among patients with *STAG2*-mutant only tumors (global log-rank *p* < 0.001). Because of the absence of events in this subgroup, four-level Cox models yielded unstable hazard estimates; prognostic effects were therefore evaluated using multivariable models including *TP53* and *STAG2* as separate covariates. (**D**) Tables showing the number of patients at risk at predefined time points for each survival analysis in panels (**A**–**C**), illustrating cohort attrition over time and supporting the interpretation of late survival differences. OS, overall survival; WT, wild-type; Mut, mutated.

To explore joint effects, patients were stratified according to combined *TP53/STAG2* mutation status (wild-type/wild-type, *TP53*-mutant only, *STAG2*-mutant only, and double-mutant) (Figure 7). Kaplan–Meier curves demonstrated the poorest OS in the *TP53*-mutant/*STAG2*-wild-type group, whereas the *STAG2*-mutant-only subgroup exhibited excellent outcomes, with no deaths observed during follow-up (global log-rank *p* < 0.001).

Because of the absence of events in this subgroup, four-level Cox proportional hazards models yielded unstable hazard ratio estimates; therefore, prognostic effects were evaluated using multivariable models with *TP53* and *STAG2* entered as separate covariates, confirming opposite and independent effects on OS. To further assess the robustness of the survival associations, we repeated the Kaplan–Meier analysis after restricting the dataset to pathogenic and likely pathogenic variants only, excluding variants of uncertain significance. The resulting survival curves are shown in Supplementary Figure S1.

In contrast to OS, no statistically significant differences in disease-free survival (DFS) were observed according to *TP53* or *FGFR3* mutation status. Kaplan–Meier analyses showed largely overlapping DFS curves between mutated and wild-type tumors for both genes (log-rank *p* = 0.10 and *p* = 0.58, respectively), and none of the examined genes retained prognostic significance for DFS in Cox regression models.

### 3.8. Comparison with TCGA-BLCA Dataset

**Figure 8:**
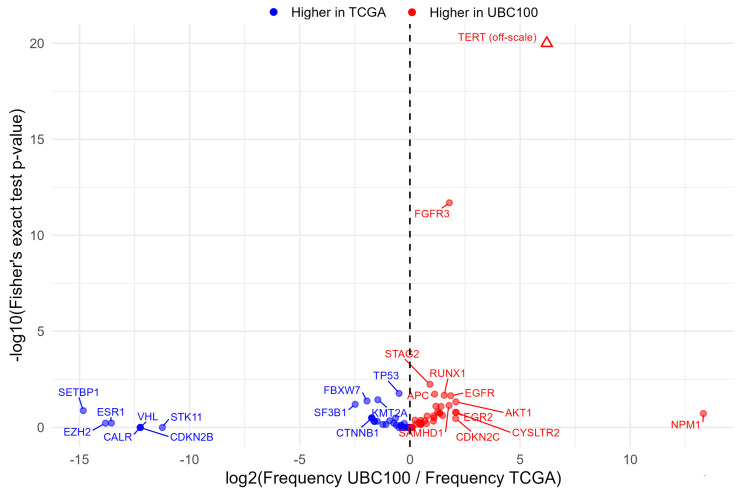
Comparative mutation frequency analysis between the UBC100 cohort and TCGA-BLCA. Volcano plot showing gene-level differences in mutation frequency between UBC100 and TCGA-BLCA cohorts, restricted to pathogenic/likely pathogenic/variants of uncertain significance (P/LP/VUS) single-nucleotide variants and small insertions/deletions in panel genes. The *x*-axis represents the log_2_ fold-change in mutation frequency (UBC100 vs. TCGA), while the *y*-axis shows −log_10_ *p*-values from Fisher’s exact tests. Positive values indicate enrichment in UBC100, whereas negative values indicate enrichment in TCGA. The *TERT* promoter mutation is shown as an off-scale point due to its exceptionally strong statistical significance. Selected recurrently altered genes are labeled for clarity.

To contextualize our findings, we compared mutation frequencies in the UBC100 cohort with publicly available TCGA bladder cancer (TCGA-BLCA) data (Figure 8).

Volcano plot analysis confirmed strong enrichment of *TERT* promoter and *FGFR3* alterations in UBC100 relative to TCGA-BLCA. In contrast, the majority of other panel genes exhibited broadly comparable mutation frequencies between the two cohorts. These differences are most plausibly explained by disparities in cohort composition, particularly the higher proportion of non–muscle-invasive tumors in UBC100 compared with the predominantly muscle-invasive disease represented in TCGA-BLCA. To evaluate whether differences between UBC100 and TCGA-BLCA were influenced by cohort composition, we repeated the mutation frequency comparison after restricting the UBC100 cohort to muscle-invasive cases only (Supplementary Figure S2). The pronounced enrichment of *TERT* promoter mutations persisted in the MIBC-restricted analysis, reflecting the targeted detection of promoter hotspot variants in the panel design.

Across other major pathways, including chromatin remodeling, WNT signaling, and DNA damage–repair, mutation frequencies were largely similar between cohorts, although a modest enrichment of DDR-related alterations was observed in UBC100. In summary, the pathway-level mutational landscape of the UBC100 cohort was largely consistent with published TCGA data while highlighting stage-associated differences and the influence of targeted panel design on observed mutation frequencies.

## 4. Discussion

A distinctive aspect of our study is the use of a broad pan-cancer sequencing panel rather than a bladder-restricted assay, enabling detection of both canonical urothelial drivers and less frequent alterations with potential biological or therapeutic relevance. In this cohort of 100 patients with urothelial bladder cancer profiled by targeted next-generation sequencing, we identified reproducible survival patterns associated with recurrent genomic alterations. These findings underscore the biological heterogeneity of the disease and support the integration of genomic features with clinicopathological assessment. The prognostic associations observed in our cohort are biologically plausible and consistent with the functional roles of the affected genes. *TP53* encodes a central tumor suppressor involved in genomic stability, cell-cycle regulation, and apoptosis, and loss-of-function alterations have repeatedly been associated with aggressive tumor behavior and adverse outcomes in urothelial carcinoma [13,14]. In our analysis, *TP53* mutations were associated with inferior overall survival and retained prognostic significance after adjustment for age and gender, suggesting that their impact reflects intrinsic tumor biology rather than demographic confounding. These findings support the potential contribution of *TP53* status to molecular risk stratification, particularly among patients otherwise classified as intermediate risk based on clinicopathological characteristics [15,16].

After *TERT* promoter alterations, *FGFR3* was the most frequently altered gene in our cohort, with pathogenic/likely pathogenic variants or variants of uncertain significance detected in 48% of patients. This frequency is consistent with most Western urothelial bladder cancer cohorts, where *FGFR3* mutations are enriched in papillary, low-grade, non–muscle-invasive tumors and are typically associated with a more indolent disease course [17]. In contrast, substantially lower frequencies have been reported in Han Chinese populations, highlighting possible population-specific differences in the molecular landscape of urothelial bladder cancer [18]. Although *FGFR3*-mutant tumors in our cohort showed a trend toward improved survival, this association did not retain independent significance in multivariable models, likely reflecting the strong correlation between *FGFR3* activation and favorable clinicopathological features such as lower grade and stage and co-occurrence with protective alterations including *STAG2*. These observations emphasize that the prognostic relevance of *FGFR3* is context-dependent and should be interpreted within broader molecular and clinical subtypes [19].

The use of a broader pan-cancer panel also enabled the detection of alterations in genes not consistently represented in bladder-focused sequencing assays. While canonical drivers such as *TP53* and *FGFR3* remain central to bladder cancer biology, extended panels allow simultaneous interrogation of additional pathways, including DNA damage response and chromatin regulation. Such broader profiling improves characterization of molecular heterogeneity and may reveal biologically or therapeutically relevant alterations not captured by restricted urothelial-specific panels. Targeted sequencing panels also reflect current clinical genomic testing practice, where high-depth analysis of predefined cancer-associated genes enables robust detection of clinically relevant alterations—including regulatory hotspots such as the *TERT* promoter—in routinely processed tumor specimens.

One of the most striking observations in our dataset was the association between *STAG2* mutations and favorable clinical outcomes. *STAG2* encodes a core cohesin subunit involved in chromatid cohesion and transcriptional regulation [20]. Although generally regarded as a tumor suppressor, previous studies have reported enrichment of *STAG2* inactivation in low-grade and early-stage urothelial carcinomas, with potential associations with reduced chromosomal instability and more indolent disease behavior [21,22,23,24], with context-dependent effects also reported [25]. Consistent with these observations, patients with *STAG2*-mutant/*TP53*-wild-type tumors in our cohort exhibited excellent survival, suggesting that *STAG2* alterations may define a favorable molecular subset. The biological basis of this association remains unclear but may reflect constrained evolutionary trajectories or early tumorigenic events that limit acquisition of high-risk alterations [26].

Our analysis also highlights the importance of considering combined mutational states. When recurrent alterations were evaluated jointly, distinct survival patterns emerged, suggesting that combinatorial genomic signatures may provide more refined prognostic stratification than single-gene markers alone. However, given the limited number of double-mutant cases, these observations should be interpreted cautiously and warrant validation in larger, prospectively collected cohorts [27,28,29].

Gene-level differences observed between NMIBC and MIBC in our cohort further support the existence of distinct molecular trajectories underlying bladder cancer progression. The predominance of *FGFR3* and *STAG2* alterations in NMIBC is consistent with a papillary, genomically more stable disease pathway, whereas enrichment of *TP53* mutations and DNA damage–repair (DDR) gene alterations in MIBC suggests increased genomic instability and impaired DNA repair capacity in invasive tumors [30]. The increased frequency of alterations in DDR-related genes, particularly *ATM* and components of the homologous recombination pathway (*BRCA1*, *BRCA2*, *PALB2*), may have therapeutic implications, although these observations require validation in larger cohorts and in the context of treatment response [31,32].

In addition to single-gene analyses, we explored patterns of co-occurrence and mutual exclusivity among frequently altered genes. As expected, a strong mutually exclusive relationship was observed between *FGFR3* and *TP53*, supporting the well-established molecular dichotomy between papillary, *FGFR3*-driven tumors and genomically unstable, *TP53*-altered lesions. Together with the divergent survival behavior observed in our cohort, these findings reinforce the view that these pathways define biologically and clinically distinct evolutionary trajectories within urothelial bladder cancer [33,34].

Our analysis also highlighted less commonly discussed relationships. In particular, *FGFR3* alterations tended to be mutually exclusive with *BRCA1* mutations. Although this relationship has not been consistently reported in prior urothelial cancer cohorts and was observed in a limited number of cases, it is biologically plausible because *FGFR3*-mutated tumors are typically associated with lower chromosomal instability, whereas *BRCA1* dysfunction promotes defective DNA repair and genomic scarring [32,35]. This observation should therefore be considered hypothesis-generating and warrants validation in larger datasets. In contrast, *RUNX1* and *PTCH1* alterations co-occurred more often than expected, although the number of double-mutant tumors was small (*n* = 3), and the finding should be interpreted cautiously, given the multiple comparisons inherent in interaction analyses.

Beyond canonical urothelial bladder cancer drivers (*TERT*, *FGFR3*, *TP53*, *STAG2*, and *PIK3CA*), our cohort demonstrated relatively frequent alterations in additional genes, including *ERBB2*, *ATM*, *KMT2A*, *TET2*, and *ASXL1*. Alterations in *ERBB2* and *ATM* may be clinically relevant, given their association with potentially actionable vulnerabilities and DNA damage response–related biology in urothelial carcinoma [36]. In contrast, recurrent alterations in epigenetic and chromatin regulators such as *KMT2A*, *TET2,* and *ASXL1* warrant cautious interpretation in tumor-only sequencing datasets, as these genes are also commonly implicated in age-related clonal hematopoiesis [37]. Although stringent variant filtering was applied, tumor-only sequencing cannot fully exclude hematopoietic contributions, particularly for low-variant-allele-frequency events. The detection of uncommon alterations such as *BRCA1* and *ALK* further illustrates the advantage of a pan-cancer sequencing approach. Although infrequent, such findings may inform eligibility for clinical trials or targeted therapeutic strategies, particularly in the context of DNA repair deficiency and receptor tyrosine kinase signaling [32].

Notably, prognostic effects in this cohort were more evident for overall survival than for disease-free survival. This discrepancy likely reflects clinical management patterns, in which early recurrences are often effectively controlled by surveillance and local interventions, delaying their impact on mortality. Consequently, genomic alterations may contribute more strongly to long-term disease progression and cancer-related death than to initial recurrence risk. Another factor that may influence disease-free survival in this cohort is the heterogeneity of adjuvant or intravesical treatments. Clinical management of patients in our cohort was performed according to contemporary European Association of Urology (EAU) guidelines and national healthcare recommendations, including the use of intravesical therapies such as bacillus Calmette–Guérin (BCG) or mitomycin C when clinically indicated. However, detailed treatment protocols were not systematically analyzed in the present study, which focused primarily on genomic alterations and their associations with clinicopathological characteristics. Consequently, potential treatment-related effects on recurrence outcomes cannot be excluded.

Our cohort included a single case of flat carcinoma in situ (pTis), a biologically aggressive but non-invasive lesion. Despite its non-invasive presentation, this tumor harbored multiple pathogenic alterations, highlighting that CIS may already acquire complex molecular features. Although this isolated case cannot support statistical inference, it highlights the potential diagnostic value of targeted sequencing in flat urothelial lesions and supports the utility of genomic profiling in early disease contexts [38].

A subset of variants initially classified as ClinVar-unmatched remained absent from ClinVar and other major databases after coordinate-based filtering and manual review, suggesting the presence of rare somatic alterations within the cohort. Manual re-evaluation also revealed that several variants flagged as ClinVar-unmatched by automated workflows already had ClinVar entries, illustrating how transcript selection, annotation variability, and reference genome differences can affect database concordance and novelty assignment. These findings highlight the importance of conservative interpretation and systematic manual verification before labeling alterations as novel. Analysis of the curated subset further demonstrated differences in biological interpretability between truncating and missense variants. Truncating and canonical splice-site alterations in *STAG2* were consistent with loss-of-function events affecting a cohesin complex tumor suppressor and support disruption of chromatin regulation as a relevant pathway in urothelial bladder cancer. In contrast, rare missense substitutions—including *STAG2* p.Gly129Arg, *TP53* p.Glu28Gln, and variants affecting *ATM* and *ERBB2*—remained classified as variants of uncertain significance across interpretation platforms. These observations illustrate how truncating variants in tumor suppressor genes often provide clearer biological signals, whereas rare missense substitutions frequently lack sufficient evidence for confident clinical interpretation.

Comparison with TCGA-BLCA data further illustrates how cohort composition influences observed genomic profiles. The volcano plot underscores that only a small subset of genes, most notably *TERT* promoter and *FGFR3*, account for the major differences between cohorts, whereas the majority of panel genes exhibit comparable mutation frequencies. The higher frequency of *FGFR3* alterations in our cohort likely reflects the enrichment of non–muscle-invasive tumors, whereas TCGA predominantly represents muscle-invasive disease [39]. Conversely, the broadly similar prevalence of *TP53* alterations across datasets underscores its central role across tumor stages. These comparisons reinforce the importance of interpreting sequencing results in the context of clinical composition and assay design.

To further evaluate whether differences between the UBC100 and TCGA-BLCA cohorts were driven by cohort composition, we repeated the mutation-frequency comparison after restricting the UBC100 dataset to muscle-invasive tumors only. In this sensitivity analysis, several discrepancies observed in the full-cohort comparison were attenuated. In particular, the difference in *FGFR3* mutation frequency largely disappeared, consistent with the known enrichment of *FGFR3* alterations in non–muscle-invasive bladder cancer. These observations support the interpretation that the higher proportion of NMIBC cases in the UBC100 cohort contributes substantially to the differences observed when comparing the two datasets.

Copy-number alterations are established contributors to bladder cancer biology, particularly deletions involving chromosome 9 and gains on chromosomes 1q and 8q [40]. The relatively modest copy-number burden observed in our series likely reflects both the clinical composition of the cohort and the fact that the targeted assay was not primarily optimized for CNV detection. Notably, the absence of detectable CNVs in the carcinoma in situ case illustrates that aggressive histological behavior can arise even in the presence of limited copy-number remodeling.

Beyond their descriptive value, the observed genotype–phenotype associations in the UBC100 cohort suggest potential translational implications for risk stratification and therapeutic decision-making, although these hypotheses remain exploratory and require prospective validation [41]. In this context, *TP53* alterations were associated with more aggressive disease behavior, whereas *STAG2* alterations—particularly in the absence of concurrent *TP53* mutations—may define biologically distinct, favorable subsets warranting further investigation. From a therapeutic perspective, the most clearly actionable findings involved *FGFR3* hotspot mutations, for which erdafitinib is FDA-approved in locally advanced or metastatic urothelial carcinoma using an FDA-approved companion diagnostic [42]. In contrast, alterations involving *PIK3CA*, *ERBB2/HER2*, and DNA damage–repair genes currently represent investigational or context-dependent opportunities, with clinical relevance influenced by tumor stage, prior therapy, and trial availability [43,44]. Collectively, these findings support the value of broad NGS profiling both for identifying established actionable alterations and for defining emerging molecular subsets as evidence continues to evolve [41,42,43]. Although this study was not designed to evaluate therapeutic interventions, the high prevalence of potentially actionable alterations illustrates how targeted sequencing—largely driven by recurrent alterations in well-established bladder cancer–relevant genes such as *FGFR3*, *PIK3CA*, and *ERBB2*—can identify a meaningful spectrum of clinically relevant genomic events in real-world urothelial bladder cancer cohorts.

Targeted next-generation sequencing using multigene pan-cancer panels has become a widely adopted strategy for molecular characterization of solid tumors, balancing sequencing depth, turnaround time, and cost relative to whole-exome or whole-genome approaches [45,46,47]. Several commercially available assays, including Thermo Fisher’s Oncomine and Illumina’s TruSight Oncology panels, are increasingly used in translational and clinical research to identify actionable variants and enable cross-study comparisons [48,49]. In this context, our panel-based profiling provides a pragmatic framework for genomic stratification of urothelial bladder cancer in a real-world clinical cohort. In parallel, multiple groups have reported customized targeted panels designed to capture recurrent driver alterations across cancer types, prioritizing clinically relevant genes and enabling robust detection of low-frequency mutations in routine specimen types [47,50,51].

Although whole-exome or whole-genome sequencing can provide broader genomic coverage, high-depth targeted panels remain widely used in clinical and translational oncology because they enable sensitive detection of clinically relevant alterations with standardized workflows, lower cost, and faster turnaround times.

While several genomic studies of urothelial bladder cancer have been published internationally, panel-based NGS data from Southeastern Europe remain limited. To our knowledge, this is the first targeted sequencing study of urothelial bladder cancer from North Macedonia and represents one of the largest clinically annotated single-center panel-based datasets from the region.

In summary, our study confirms the adverse prognostic impact of *TP53* alterations, highlights *STAG2* as a marker of highly favorable outcome, and demonstrates that gene–gene interactions reveal clinically meaningful heterogeneity not captured by single-gene analyses alone. These findings support the integration of genomic features into biologically informed risk stratification models for urothelial bladder cancer.

This study has several limitations. First, it was conducted at a single tertiary center, which may limit generalizability. Second, the retrospective design introduces potential selection bias and incomplete follow-up, although clinical data were independently verified. Third, the cohort size limited statistical power for certain subgroup analyses, particularly for disease-free survival. Accordingly, these findings should be regarded as hypothesis-generating and warrant confirmation in larger, prospective cohorts. An additional limitation is that sequencing was performed on tumor tissue without matched normal samples; therefore, rare germline variants cannot be completely excluded for certain genes, particularly those associated with hereditary cancer predisposition such as *BRCA1*/*2* and *ATM*. Tumor-only sequencing has limited ability to distinguish somatic from rare germline variants, and confirmatory germline testing may be required when clinically indicated [52].

Importantly, the genomic contrasts observed in this study were detectable using targeted sequencing data restricted to P/LP/VUS somatic variants, without reliance on tumor mutational burden (TMB) metrics. TMB was not calculated in the present analysis because the targeted sequencing panel used in this study spans approximately 0.2 Mb of genomic territory across 95 genes, which is substantially below the size generally considered necessary for reliable panel-based TMB estimation (typically ≥1 Mb) [53,54]. Consequently, TMB values derived from this assay would likely be unstable and potentially misleading. This consideration is consistent with current methodological recommendations for the interpretation of targeted NGS data in cancer genomic profiling. Overall, these findings are consistent with previously reported targeted sequencing approaches and established clinical and interpretative frameworks for urothelial carcinoma [38,55,56,57,58].

## 5. Conclusions

Using a broad pan-cancer targeted next-generation sequencing panel, we characterized the genomic landscape of urothelial bladder cancer in a clinically annotated real-world cohort of 100 patients. This approach captured both canonical urothelial drivers and less frequent genomic alterations that may be overlooked by bladder-restricted assays. *TP53* and *STAG2* emerged as independent prognostic markers for overall survival, whereas *FGFR3* mutations were associated with a trend toward more favorable outcomes. Notably, *STAG2*—which is not consistently represented in many bladder-focused sequencing panels—identified a molecular subset with particularly favorable survival, highlighting the added value of broader panel-based profiling. Patterns of gene co-occurrence further supported the presence of biologically distinct molecular pathways, while the overall burden of copy-number alterations was modest. Collectively, these findings support the integration of genomic features into biologically informed risk stratification strategies for urothelial bladder cancer. Validation in larger independent cohorts will be required to define the clinical utility of these genomic markers for risk-adapted clinical management.

## Figures and Tables

**Table 1:** Clinicopathological and demographic characteristics of the study population at initial TURBT.

Characteristics	Values ^1^
Gender, *n* (%)	
Male	78 (78.0)
Female	22 (22.0)
Smoking history, *n* (%)	
Never/rare smoker	44 (44.0)
Heavy or moderate smoker	56 (56.0)
Age (years), mean [range]	66.6 [41–86]
≤70, *n* (%)	62 (62.0)
>70, *n* (%)	38 (38.0)
Pathological T stage ^2^, *n* (%)	
pTis	1 (1.0)
pTa	36 (36.0)
pT1	30 (30.0)
pT2	33 (33.0)
Histological grade ^2^, *n* (%)	
Low	30 (30.0)
High	70 (70.0)
Tumor focality ^2^, *n* (%)	
CIS flat lesion	1 (1.0)
Unifocal	70 (70.0)
Multifocal	30 (30.0)
Tumor size ^2^, *n* (%)	
≤3 cm	40 (40.0)
>3 cm	60 (60.0)

^1^ Values are presented as numbers (%) or mean [range]. ^2^ At the time of initial diagnosis.

**Table 2:** Candidate rare or ClinVar-unmatched substitution variants.

Gene	Substitution	Variant Classification	Coding Region Change	Amino Acid Change	Clinical Significance
*STAG2*	G>A	Splice site	NM_001042750.2:c.462+1G>A		LP
*STAG2*	C>T	Nonsense	NM_001042750.2:c.499C>T	p.Gln167*	LP
*STAG2*	G>T	Nonsense	NM_001042750.2:c.1024G>T	p.Glu342*	LP
*STAG2*	C>G	Nonsense	NM_001042750.2:c.1958C>G	p.Ser653*	LP
*TP53*	C>G	Missense	NM_000546.5:c.82G>C	p.Glu28Gln	VUS
*ATM*	T>C	Missense	NM_000051.3:c.1988T>C	p.Leu663Ser	VUS
*ATM*	G>A	Missense	NM_000051.3:c.3556G>A	p.Glu1186Lys	VUS
*ATM*	A>G	Splice site	NM_000051.3:c.6452+3A>G		VUS
*ERBB2*	G>A	Missense	NM_004448.3:c.308G>A	p.Arg103Gln	VUS
*STAG2*	G>A	Missense	NM_001042750.2:c.385G>A	p.Gly129Arg	VUS

Variants shown represent a manually curated subset of substitution-type events (SNP/DNP/TNP/ONP) that were initially unmatched in ClinVar by GRCh37/hg19 coordinate-based screening and prioritized from frequently altered genes in the UBC100 cohort (*TERT* promoter, *FGFR3*, *TP53*, *STAG2*, *ATM*, and *ERBB2*). ClinVar status was verified using ClinVar and external interpretation platforms (Franklin/Genoox, GeneBe, and VarSome). “Unmatched” indicates the absence of a ClinVar record at the time of manual final preparation. TCGA, The Cancer Genome Atlas; CIViC, clinical interpretation of variants in cancer; COSMIC, catalog of somatic mutations in cancer. * indicates a nonsense (stop-gain) mutation resulting in a truncated protein. LP, likely pathogenic; VUS, variant of unknown significance.

**Table 3:** Association between clinicopathological characteristics and mutation status of recurrently altered genes in the UBC100 cohort.

Parameter	*TERT*	*FGFR3*	*TP53*	*STAG2*
**Smoking history** (never-rare vs. moderate-heavy)	OR = 0.976 95% CI 0.367–2.634 *p* = 1.000	OR = 2.153 95% CI 0.898–5.291 *p* = 0.071	OR = 0.724 95% CI 0.287–1.785 *p* = 0.530	OR = 1.884 95% CI 0.708–5.120 *p* = 0.179
	*q* = 1.000	*q* = 1.000	*q* = 1.000	*q* = 1.000
**Tumor size** (>3 cm vs. ≤3 cm)	OR = 0.780 95% CI 0.291–2.114 *p* = 0.648	OR = 2.251 95% CI 0.924–5.659 *p* = 0.066	OR = 0.289 95% CI 0.098–0.776 ***p* = 0.010**	OR = 1.933 95% CI 0.722–5.238 *p* = 0.171
	*q* = 1.000	*q* = 1.000	*q* = 0.304	*q* = 1.000
**Primary tumor stage** (pTis/pTa/pT1/pT2/)	*p* = 0.519	***p*** ** = 0.00071**	***p*** ** = 0.0000091**	***p*** ** = 0.022**
	*q* = 1.000	***q*** ** = 0.0353**	***q*** ** = 0.00123**	*q* = 0.596
**Invasiveness** (NMIBC vs. MIBC)	OR = 0.635 95% CI 0.200–1.833 *p* = 0.474	OR = 5.011 95% CI 1.880–14.430 ***p* = 0.00054**	OR = 0.108 95% CI 0.036–0.300 ***p* = 0.0000015**	OR = 3.745 95% CI 1.115–16.437 ***p* = 0.030**
	*q* = 1.000	***q*** ** = 0.0324**	***q*** ** = 0.00046**	*q* = 0.736
**Histological grade** (Low vs. High)	OR = 0.644 95% CI 0.231–1.855 *p* = 0.461	OR = 7.347 95% CI 2.387–27.557 ***p* = 0.000068**	OR = 0.127 95% CI 0.023–0.470 ***p* = 0.00028**	OR = 3.777 95% CI 1.351–10.828 ***p* = 0.006**
	*q* = 1.000	***q*** ** = 0.0067**	***q*** ** = 0.020**	*q* = 0.209
**Recurrence** (Yes vs. No)	OR = 0.914 95% CI 0.233–4.388 *p* = 1.000	OR = 0.871 95% CI 0.238–3.186 *p* = 1.000	OR = 3.879 95% CI 0.788–37.861 *p* = 0.079	OR = 1.608 95% CI 0.381–6.060 *p* = 0.518
	*q* = 1.000	*q* = 1.000	*q* = 1.000	*q* = 1.000

Association analysis between clinicopathological parameters and mutation status of recurrently altered genes (*TERT*, *FGFR3*, *TP53*, and *STAG2*) in the UBC100 cohort. Associations were evaluated using Fisher’s exact test for binary variables (2 × 2 tables) or Fisher’s exact test for multi-category variables (R × C tables), as appropriate. For binary comparisons, odds ratios (ORs) with 95% confidence intervals (CI) and corresponding *p*-values are reported; for multi-category variables, *p*-values are shown. q-values were computed using Benjamini–Hochberg FDR correction across all gene–clinic association tests (*n* = 315). Odds ratios are reported relative to the reference category indicated in parentheses. NMIBC, non–muscle-invasive bladder cancer; MIBC, muscle-invasive bladder cancer; OR, odds ratio; CI, confidence interval. Bold values indicate statistically significant associations (*p*/*q* < 0.05).

## Data Availability

The data supporting the findings of this study are available upon reasonable request from the corresponding author, Sasho Panov (sasho@mt.net.mk).

## Supplementary Materials

The following supporting information can be downloaded at https://www.mdpi.com/article/10.3390/cancers18061026/s1, Supplementary Table S1: Targeted panel gene list. Supplementary Table S2: Comparison of representative urothelial bladder cancer–focused sequencing panels and the pan-cancer panel used in this study. Supplementary Table S3: Recurrent pathogenic, likely pathogenic, and variants of uncertain significance (P/LP/VUS) in key driver genes identified in the UBC100 cohort. Supplementary Table S4: Clinically actionable somatic alterations in the UBC100 cohort. Supplementary Figure S1: Kaplan–Meier overall survival according to *TP53* and *STAG2* mutational status (pathogenic and likely pathogenic variants only). Survival analyses were repeated after excluding variants of uncertain significance (VUS). (A) Overall survival stratified by *TP53* mutation status. (B) Overall survival stratified by *STAG2* mutation status. (C) Overall survival according to combined *TP53*×*STAG2* mutation status. (D) Corresponding numbers at risk for panels (A–C). *p*-values were calculated using the log-rank test. Association remained statistically significant, confirming that the prognostic effects are not driven by inclusion of VUS variants. Supplementary Figure S2: Comparative mutation frequency analysis between muscle-invasive bladder cancer cases in the UBC100 cohort and the TCGA-BLCA dataset. Volcano plot showing gene-level differences in mutation frequency between the UBC100 cohort restricted to muscle-invasive bladder cancer (MIBC) cases and the TCGA-BLCA cohort, using the same filtering criteria as the primary analysis. The analysis includes pathogenic/likely pathogenic/variants of uncertain significance (P/LP/VUS) single-nucleotide variants and small insertions/deletions in panel genes. The x-axis represents the log_2_ fold-change in mutation frequency (UBC100 MIBC vs. TCGA), while the y-axis shows −log_10_ *p*-values from Fisher’s exact tests. Positive values indicate enrichment in the UBC100 MIBC subset, whereas negative values indicate enrichment in TCGA. The *TERT* promoter mutation is shown as an off-scale point due to its exceptionally strong statistical significance. Selected recurrently altered genes are labeled for clarity.

## Abbreviations

The following abbreviations are used in this manuscript:

UBC	Urothelial bladder cancer
SNV	Single nucleotide variation (alteration)
CNA	Copy-number alteration (variation)
NMIBC	Non-muscle-invasive bladder cancer
MIBC	Muscle-invasive bladder cancer
TURBT	Transurethral resection of the bladder tumor
VAF	Variant allele frequency
CIS	Carcinoma in situ
OS	Overall survival
DFS	Disease-free survival
PCR	Polymerase chain reaction
*FGFR3*	Fibroblast growth factor receptor 3
*HRAS*	Harvey rat sarcoma
*KRAS*	Kirsten rat sarcoma virus
*PIK3CA*	Phosphatidylinositol-4,5-bisphosphate 3-kinase catalytic subunit alpha
*STAG2*	Cohesin subunit SA-2
*TERT*	Telomerase reverse transcriptase
*TP53*	Tumor protein 53
