# Supplementary material for: Pan-Cancer Targeted Sequencing Reveals Genomic Heterogeneity and Prognostic Subgroups in Urothelial Bladder Cancer
Source: Cancers (Basel). 2026 Mar 22;18(6):1026. doi: 10.3390/cancers18061026 (PMC13025778; doi:10.3390/cancers18061026)
Supplement: Supplementary file 1 [file cancers-18-01026-s001.zip › Supplementary Figure S1.pdf]

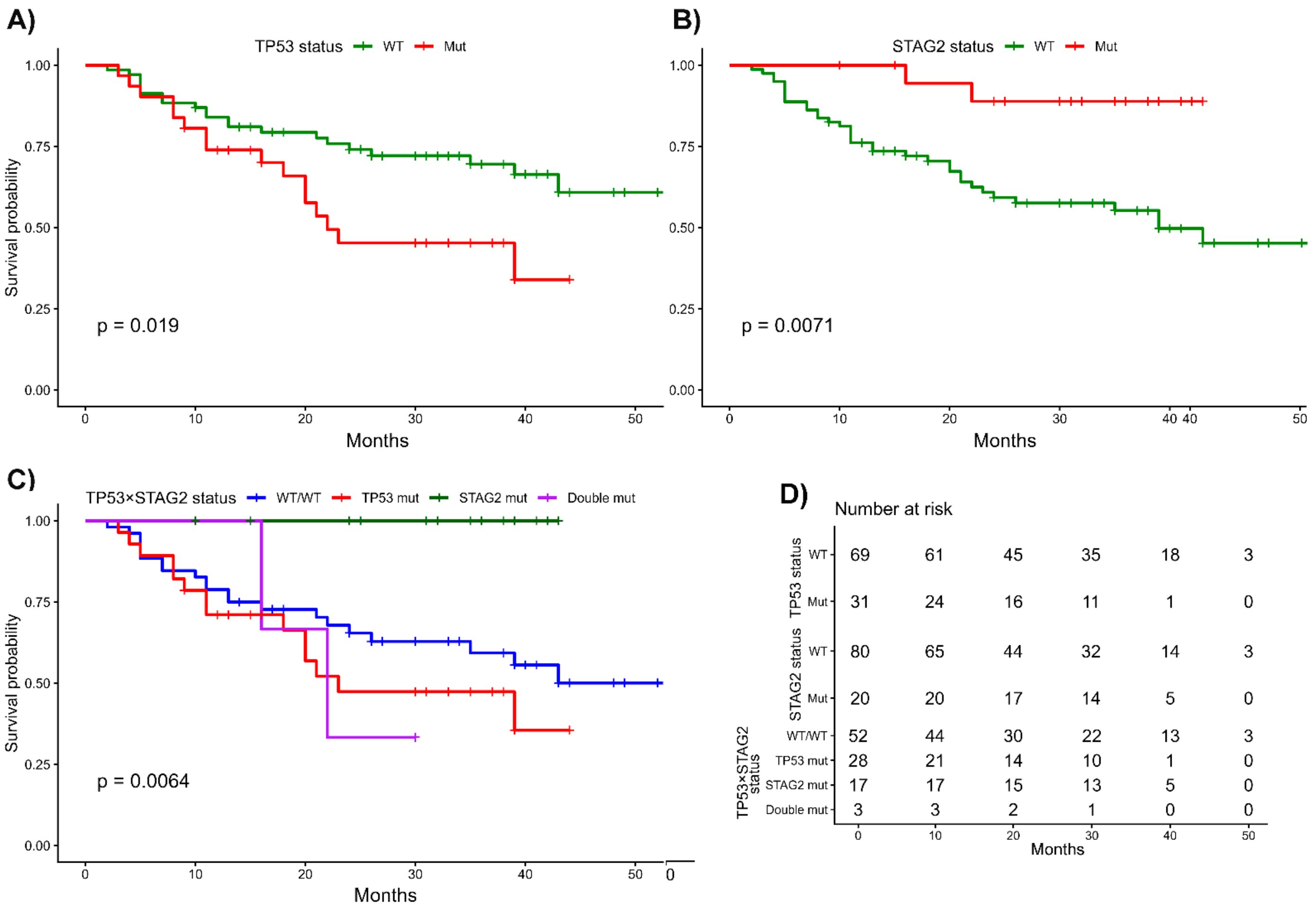

Supplementary Figure S1. Kaplan–Meier overall survival according to TP53 and STAG2 mutational status (pathogenic and likely pathogenic variants only). Survival analyses were repeated after excluding variants of uncertain significance (VUS). **(A)** Overall survival stratified by TP53 mutation status. **(B)** Overall survival stratified by STAG2 mutation status. **(C)** Overall survival according to combined TP53×STAG2 mutation status. **(D)** Corresponding numbers at risk for panels A–C. P-values were calculated using the log-rank test. Association remained statistically significant, confirming that the prognostic effects are not driven by inclusion of VUS variants.
