# Supplementary material for: Pan-Cancer Targeted Sequencing Reveals Genomic Heterogeneity and Prognostic Subgroups in Urothelial Bladder Cancer
Source: Cancers (Basel). 2026 Mar 22;18(6):1026. doi: 10.3390/cancers18061026 (PMC13025778; doi:10.3390/cancers18061026)
Supplement: Supplementary file 1 [file cancers-18-01026-s001.zip › Supplementary Figure S2.pdf]

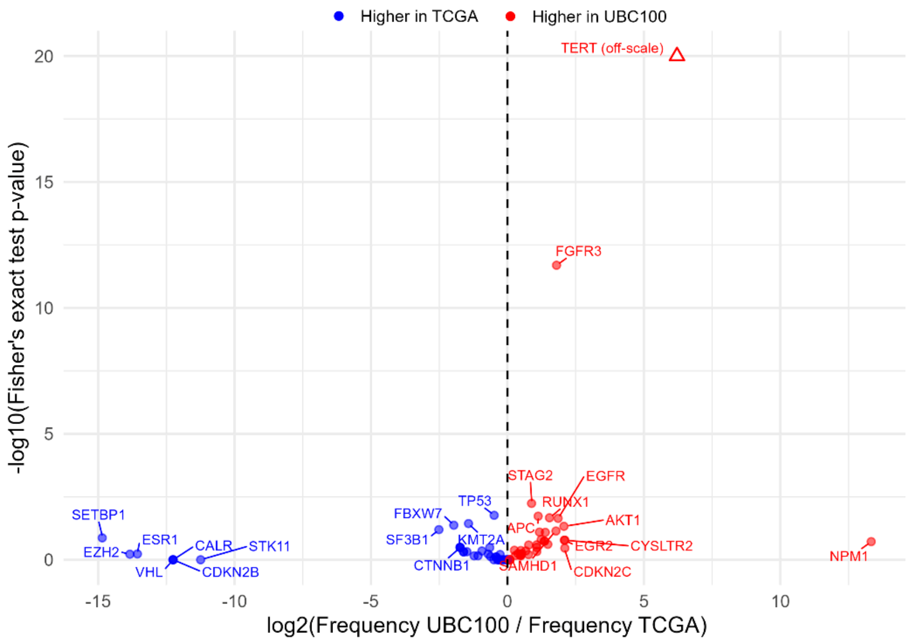

Supplementary Figure S2. Comparative mutation frequency analysis between muscle-invasive bladder cancer cases in the UBC100 cohort and the TCGA-BLCA dataset. Volcano plot showing gene-level differences in mutation frequency between the UBC100 cohort restricted to muscle-invasive bladder cancer (MIBC) cases and the TCGA-BLCA cohort, using the same filtering criteria as the primary analysis. The analysis includes pathogenic/likely pathogenic/variants of uncertain significance (P/LP/VUS) single-nucleotide variants and small insertions/deletions in panel genes. The x-axis represents the $\log_2$ fold-change in mutation frequency (UBC100 MIBC vs TCGA), while the y-axis shows $-\log_{10}$ p-values from Fisher's exact tests. Positive values indicate enrichment in the UBC100 MIBC subset, whereas negative values indicate enrichment in TCGA. The TERT promoter mutation is shown as an off-scale point due to its exceptionally strong statistical significance. Selected recurrently altered genes are labeled for clarity.
