# Supplementary material for: Pan-Cancer Targeted Sequencing Reveals Genomic Heterogeneity and Prognostic Subgroups in Urothelial Bladder Cancer
Source: Cancers (Basel). 2026 Mar 22;18(6):1026. doi: 10.3390/cancers18061026 (PMC13025778; doi:10.3390/cancers18061026)
Supplement: Supplementary file 1 [file cancers-18-01026-s001.zip › Supplementary Table S1.pdf]

Supplementary Table S1: Targeted panel gene list

No.	Chr.	HUGO	Region (Exon/Intron/Promoter)	NM	OMIM
1	9	*ABL1*	exon 4- 10	NM_007313.3	189980
2	14	*AKT1*	exon 3- 15	NM_001382430.1	164730
3	2	*ALK*	exon 21- 28, intron 20	NM_004304.4	105590
4	5	*APC*	exon 2- 16	NM_000038.5	611731
5	20	*ASXL1*	exon 12, 13	NM_015338.5	612990
6	11	*ATM*	exon 2- 63	NM_000051.3	607585
7	3	*BAP1*	exon 1- 17	NM_004656.3	603089
8	X	*BCOR*	exon 2- 15	NM_001123385.1	300485
9	11	*BIRC3*	exon 2- 9	NM_001165.4	601721
10	7	*BRAF*	exon 1- 18	NM_004333.4	164757
11	17	*BRCA1*	exon 2- 23	NM_007294.4	113705
12	13	*BRCA2*	exon 2- 27	NM_000059.3	600185
13	X	*BTK*	exon 15	NM_000061.2	300300
14	19	*CALR*	exon 9	NM_004343.3	109091
15	11	*CBL*	exon 8, 9	NM_005188.3	165360
16	9	*CDKN2A*	exon 1- 3	NM_000077.5	600160
17	9	*CDKN2B*	exon 1, 2	NM_004936.3	600431
18	1	*CDKN2C*	exon 1, 2	NM_078626.2	603369
19	19	*CEBPA*	exon 1	NM_004364.4	116897
20	1	*CSF3R*	exon 14, 17	NM_000760.4	138971
21	3	*CTNNB1*	exon 3	NM_001904.3	116806
22	13	*CYSLTR2*	exon 5	NM_001308476.3	605666
23	1	*DDR2*	exon 3- 18	NM_006182.4	191311
24	14	*DICER1*	exon 2- 27	NM_177438.3	606241
25	2	*DNMT3A*	exon 2- 23	NM_022552.4	602769
26	1	*DPYD*	exon 11, 13, 14, 22, intron 10 (partial)	NM_000110.4	612779
27	7	*EGFR*	exon 1- 28	NM_005228.4	131550
28	10	*EGR2*	exon 2, 3	NM_000399.4	129010
29	X	*EIF1AX*	exon 1, 2	NM_001412.4	300186
30	17	*ERBB2*	exon 1- 27	NM_004448.3	164870
31	6	*ESR1*	exon 4- 8	NM_000125.4	133430
32	12	*ETV6*	exon 1- 8	NM_001987.5	600618
33	7	*EZH2*	exon 2- 20	NM_004456.4	601573
34	4	*FBXW7*	exon 4- 14	NM_001349798.2	606278
35	8	*FGFR1*	exon 4-7, 11-16	NM_023110.3	136350
36	10	*FGFR2*	exon 5- 9, 12- 18	NM_000141.5	176943
37	4	*FGFR3*	exon 3, 6-18	NM_000142.5	134934
38	5	*FGFR4*	exon 3, 6, 9, 10, 12, 13, 15, 16	NM_213647.3	134935
39	13	*FLT3*	exon 10, 11, 14-17, 20	NM_004119.3	136351
40	19	*GNA11*	exon 5	NM_002067.4	139313
41	9	*GNAQ*	exon 5	NM_002072.4	600998
42	20	*GNAS*	exon 8, 9	NM_000516.7	139320
43	1	*H3F3A*	exon 2	NM_002107.4	601128
44	11	*HRAS*	exon 2, 3	NM_005343.4	190020
45	2	*IDH1*	exon 4	NM_005896.3	147700
46	15	*IDH2*	exon 4	NM_002168.3	147650
47	1	*JAK1*	exon 3- 20, 22- 24	NM_002227.4	147795
48	9	*JAK2*	exon 8, 12- 16	NM_004972.3	147796
49	19	*JAK3*	exon 13	NM_000215.3	600173
50	19	*KEAP1*	exon 2- 5	NM_203500.2	606016
51	4	*KIT*	exon 8- 20	NM_000222.2	164920
52	11	*KMT2A*	exon 1- 20	NM_001197104.2	159555
53	12	*KRAS*	exon 2, 3, 4	NM_004985.5	190070
54	15	*MAP2K1*	exon 1- 9	NM_002755.4	176872
55	10	*MAP3K8*	intron 8	NM_005204.3	191195
56	7	*MET*	exon 2- 21, intron 7, 13, 14	NM_000245.4	164860
57	1	*MPL*	exon 10	NM_005373.2	159530
58	6	*MYB*	exon 1- 16	NM_001130173.2	189990
59	8	*MYBL1*	exon 1- 16	NM_001080416.4	159405
60	3	*MYD88*	exon 2- 5	NM_002468.4	602170

61	9	*NOTCH1*	exon 26, 27, 28, 31, 34	NM_017617.4	190198
62	1	*NOTCH2*	exon 26, 27, 31, 34	NM_024408.3	600275
63	5	*NPM1*	exon 1- 11	NM_002520.7	164040
64	1	*NRAS*	exon 2, 3, 4	NM_002524.4	164790
65	16	*PALB2*	exon 1- 13	NM_024675.4	610355
66	4	*PDGFRA*	exon 12, 14, 18	NM_006206.5	173490
67	3	*PIK3CA*	exon 8, 10, 21	NM_006218.3	171834
68	20	*PLCB4*	exon 24	NM_001377142.1	600810
69	16	*PLCG2*	exon 19, 20, 24	NM 002661.4	600220
70	19	*POLD1*	exon 7, 11, 14	NM_002691.4	174761
71	12	*POLE*	exon 9, 11, 13, 14	NM_006231.4	174762
72	7	*POT1*	exon 5- 19	NM_015450.2	606478
73	9	*PTCH1*	eon 1- 23	NM_000264.5	601309
74	10	*PTEN*	exon 1- 9	NM_000314.6	601728
75	12	*PTPN11*	exon 3, 13	NM_002834.4	176876
76	10	*RET*	exon 1- 20, intron 7-11	NM_020975.6	164761
77	6	*ROS1*	exon 2, 11, 16, 17, 23, 24, 28, 35-43, intron 32, 33, 34, 35, 36	NM_001378902.1	165020
78	21	*RUNX1*	exon 2- 9	NM_001754.4	151385
79	20	*SAMHD1*	exon 1- 16	NM_015474.3	606754
80	18	*SETBP1*	exon 4	NM_015559.2	611060
81	2	*SF3B1*	exon 13- 16, 18	NM_012433.3	605590
82	18	*SMAD4*	exon 2- 12	NM_005359.6	600993
83	19	*SMARCA*	exon 8, 15- 25, 30- 33	NM_003072.5	603254
84	22	*SMARCB*	exon 1- 9	NM_003073.5	601607
85	17	*SRSF2*	exon 1, 2	NM_001195427.1	600813
86	X	*STAG2*	exon 3- 35	NM_001042750.2	300826
87	17	*STAT3*	exon 5- 11, 13, 19- 23	NM_139276.3	102582
88	17	*STAT5B*	exon 15- 17	NM_012448.3	604260
89	19	*STK11*	exon 1- 9	NM_000455.4	602216
90	5	*TERT*	promoter	NM_198253.2	187270
91	4	*TET2*	exon 3- 11	NM_001127208.2	612839
92	17	*TP53*	exon 2- 11	NM_000546.5	191170
93	21	*U2AF1*	exon 2, 5, 6	NM_006758.2	191317
94	3	*VHL*	exon 1- 3	NM_000551.4	608537
95	2	*XPO1*	exon 14- 16	NM_003400.3	602559

Gene symbols follow HUGO Gene Nomenclature Committee (HGNC) recommendations. Chromosomal locations are based on the human reference genome (hg19). Targeted regions indicate exons, introns, or promoter regions included in the sequencing panel design.

Chr., chromosome; HUGO ID, approved gene symbol according to HGNC; NM, RefSeq mRNA accession number; OMIM, Online Mendelian Inheritance in Man identifier.
