# Supplementary material for: Pan-Cancer Targeted Sequencing Reveals Genomic Heterogeneity and Prognostic Subgroups in Urothelial Bladder Cancer
Source: Cancers (Basel). 2026 Mar 22;18(6):1026. doi: 10.3390/cancers18061026 (PMC13025778; doi:10.3390/cancers18061026)
Supplement: Supplementary file 1 [file cancers-18-01026-s001.zip › Supplementary Table S2.pdf]

Supplementary Table S2: Comparison of representative urothelial bladder cancer–focused sequencing panels and the pan-cancer panel used in this study.

Gene / Pathway category	Representative genes	Typical bladder-focused panels	Pan-cancer panel used in this study	Example relevance in UBC100
Canonical urothelial drivers	FGFR3, TP53, PIK3CA, HRAS	✓	✓	Major recurrent drivers detected in the cohort
Chromatin / cohesin regulation	STAG2, KMT2A, ARID1A	Variable / often absent	✓	STAG2 mutations associated with favorable clinical outcomes
DNA damage response (DDR)	ATM, BRCA1, BRCA2	Variable	✓	Detection of alterations in DDR pathway genes
Receptor tyrosine kinase signaling	ERBB2, EGFR, MET	Variable	✓	Additional signaling alterations identified
Additional oncogenic pathways	KRAS, NRAS, BRAF	Rare	✓	Expanded molecular characterization of tumors

The table illustrates differences in pathway coverage between typical bladder-focused targeted sequencing panels and the broader pan-cancer panel used in the present study. Representative bladder-focused sequencing panels include previously published targeted assays designed for urothelial cancer profiling [38,55,56], which primarily focus on canonical urothelial driver genes.

Commercial assays such as the Nonacus bladder cancer panel follow similar design principles and target a limited set of recurrent bladder cancer genes (https://nonacus.com/wp-content/uploads/2023/08/Nonacus_cell3_target_Bladder_Cancer_Datasheet_v1.pdf, accessed on 06 March 2026).
