# Supplementary material for: Pan-Cancer Targeted Sequencing Reveals Genomic Heterogeneity and Prognostic Subgroups in Urothelial Bladder Cancer
Source: Cancers (Basel). 2026 Mar 22;18(6):1026. doi: 10.3390/cancers18061026 (PMC13025778; doi:10.3390/cancers18061026)
Supplement: Supplementary file 1 [file cancers-18-01026-s001.zip › Supplementary Table S3.pdf]

Supplementary Table S3: Recurrent pathogenic, likely pathogenic, and variants of uncertain significance (P/LP/VUS) in key driver genes identified in the UBC100 cohort

Gene	Transcript	HGVS_c	HGVS_p	Tumors (n)	Total variants per gene
TERT	NM_198253.2	c.-124C>T	/ (promoter)	60	74
	NM_198253.2	c.-146C>T	/ (promoter)	11	
	NM_198253.2	c.-54C>A	/ (promoter)	1	
	NM_198253.2	c.-57A>C	/ (promoter)	1	
	NM_198253.2	c.-212C>A	/ (promoter)	1	
FGFR3	NM_000142.5	c.746C>G	p.Ser249Cys	27	52
	NM_000142.5	c.1118A>G	p.Tyr373Cys	6	
	NM_000142.5	c.1150T>C	p.Phe384Leu	5	
	NM_000142.5	c.742C>T	p.Arg248Cys	5	
	NM_000142.5	c.1108G>T	p.Gly370Cys	4	
	NM_000142.5	c.1345C>T	p.Pro449Ser	3	
	NM_000142.5	c.1948A>G	p.Lys650Glu	2	
TP53	NM_000546.5	c.524G>A	p.Arg175His	4	28
	NM_000546.5	c.637C>T	p.Arg213Ter	4	
	NM_000546.5	c.844C>T	p.Arg282Trp	3	
	NM_000546.5	c.817C>T	p.Arg273Cys	3	
	NM_000546.5	c.743G>A	p.Arg248Gln	3	
	NM_000546.5	c.818G>A	p.Arg273His	3	
	NM_000546.5	c.1024C>T	p.Arg342Ter	2	
	NM_000546.5	c.659A>G	p.Tyr220Cys	2	
	NM_000546.5	c.455C>T	p.Pro152Leu	2	
	NM_000546.5	c.817C>A	p.Arg273Ser	2	
PIK3CA	NM_006218.3	c.1633G>A	p.Glu545Lys	9	25
	NM_006218.3	c.1624G>A	p.Glu542Lys	8	
	NM_006218.3	c.3140A>G	p.His1047Arg	5	
	NM_006218.3	c.1633G>C	p.Glu545Gln	2	
	NM_006218.3	c.3140A>T	p.His1047Leu	1	
STAG2	NM_006603.3	c.3306C>A	p.Tyr1102Ter	3	19
	NM_006603.3	c.3148C>T	p.Arg1050Ter	2	
	NM_006603.3	c.930G>A	p.Trp310Ter	2	
	NM_006603.3	c.1903C>T	p.Arg635Ter	2	
	NM_006603.3	c.1534C>T	p.Gln512Ter	2	
	NM_006603.3	c.2137C>T	p.Arg713Ter	2	
	NM_006603.3	c.4777del	p.Gln1593ArgfsTer2	2	
	NM_006603.3	c.5880_5881del	p.Glu1960AspfsTer13	2	
	NM_006603.3	c.5263C>T	p.Arg1755Ter	2	
APC	NM_000038.6	c.4348C>T	p.Arg1450Ter	3	15
	NM_000038.6	c.646C>T	p.Arg216Ter	2	
	NM_000038.6	c.3403C>T	p.Arg1135Ter	2	
	NM_000038.6	c.3181C>T	p.Gln1061Ter	2	
	NM_000038.6	c.3260T>G	p.Leu1087Ter	2	
	NM_000038.6	c.4621C>T	p.Gln1541Ter	2	
	NM_000038.6	c.4660C>T	p.Arg1554Ter	2	

ATM	NM_000051.4	c.2376G>A	p.Trp792Ter	2	12
	NM_000051.4	c.8734A>G	p.Lys2912Glu	2	
	NM_000051.4	c.2605G>A	p.Gly869Arg	2	
	NM_000051.4	c.7757G>A	p.Arg2586His	2	
	NM_000051.4	c.596C>G	p.Ser199Cys	2	
	NM_000051.4	c.1810C>T	p.Pro604Ser	2	
BRCA2	NM_000059.3	c.6475A>G	p.Asn2159Asp	2	6
	NM_000059.3	c.6591_6592del	p.Ile2198SerfsTer8	2	
	NM_000059.3	c.8187G>T	p.Lys2729Asn	2	
ERBB2	NM_004448.4	c.929C>T	p.Ser310Phe	2	5
	NM_004448.4	c.3082G>A	p.Val1028Met	1	
	NM_004448.4	c.2264G>A	p.Arg755Gln	1	
	NM_004448.4	c.368C>T	p.Ser123Phe	1	

Variant frequencies indicate the number of tumors harboring each alteration.
HGVS nomenclature follows RefSeq transcript annotations.
