# Supplementary material for: Pan-Cancer Targeted Sequencing Reveals Genomic Heterogeneity and Prognostic Subgroups in Urothelial Bladder Cancer
Source: Cancers (Basel). 2026 Mar 22;18(6):1026. doi: 10.3390/cancers18061026 (PMC13025778; doi:10.3390/cancers18061026)
Supplement: Supplementary file 1 [file cancers-18-01026-s001.zip › Supplementary Table S4.pdf]

**Supplementary Table S4: Clinically actionable somatic alterations in the UBC100 cohort**

| Gene | HGVS variant | Variant class | Clin. Sig. | n | Tier | Targeted therapy |
|---|---|---|---|---|---|---|
| ***FGFR3*** | NM_000142.5(*FGFR3*):c.746C>G (p.Ser249Cys) | Missense | P | 27 | Tier I, Level A | Erdafitinib (FGFR inhibitor) |
| | NM_000142.5(*FGFR3*):c.1118A>G (p.Tyr373Cys) | Missense | P | 6 | Tier I, Level A | |
| | NM_000142.5(*FGFR3*):c.742C>T (p.Arg248Cys) | Missense | P | 5 | Tier I, Level A | |
| | NM_000142.5(*FGFR3*):c.1108G>T (p.Gly370Cys) | Missense | LP | 4 | Tier I, Level A | |
| | NM_000142.5(*FGFR3*):c.1948A>G (p.Lys650Glu) | Missense | P | 2 | Tier II, Level D | |
| | NM_000142.5(*FGFR3*): c.1107_1108delinsTT (p.Gly370Cys) | Missense | P | 1 | Tier I, Level A | |
| ***PIK3CA*** | NM_006218.3(*PIK3CA*):c.1633G>A (p.Glu545Lys) | Missense | P | 9 | Tier II, Level C | *PI3K* pathway inhibitors (class) |
| | NM_006218.3(*PIK3CA*):c.1624G>A (p.Glu542Lys) | Missense | P | 8 | Tier II, Level C | |
| | NM_006218.3(*PIK3CA*):c.3140A>G (p.His1047Arg) | Missense | P | 5 | Tier II, Level C | |
| | NM_006218.3(*PIK3CA*):c.1633G>C (p.Glu545Gln) | Missense | LP | 1 | Tier II, Level C | |
| | NM_006218.3(*PIK3CA*):c.1633G>C (p.Glu545Gln) | Missense | P | 1 | Tier II, Level C | |
| | NM_006218.3(*PIK3CA*):c.3140A>T (p.His1047Leu) | Missense | P | 1 | Tier II, Level C | |
| ***ERBB2*** | *ERBB2* | CNV Amp | | 5 | Tier II, Level C | HER2-directed therapies (e.g., ADCs) |
| | NM_004448.3(*ERBB2*):c.929C>T (p.Ser310Phe) | Missense | LP | 4 | Tier II, Level C | |
| | NM_004448.3(*ERBB2*):c.929C>A (p.Ser310Tyr) | Missense | P | 2 | Tier II, Level C | |

Clinically actionable somatic alterations identified in the UBC100 cohort, classified according to AMP/ASCO/CAP joint consensus recommendations for the interpretation of sequence variants in cancer (Tier I–IV). Approved targeted therapies (e.g., erdafitinib for *FGFR3*-altered urothelial carcinoma) are indicated where applicable based on current regulatory approvals [57,58]. HGVS, Human Genome Variation Society nomenclature; Clin. Sig., clinical significance according to ClinVar; P, pathogenic; LP, likely pathogenic; VUS, variant of uncertain significance.
